# Tandem Mass Tag-Based Quantitative Proteomics Reveals Implication of a Late Embryogenesis Abundant Protein (BnLEA57) in Seed Oil Accumulation in *Brassica napus* L.

**DOI:** 10.3389/fpls.2022.907244

**Published:** 2022-06-02

**Authors:** Zhongjing Zhou, Baogang Lin, Jinjuan Tan, Pengfei Hao, Shuijin Hua, Zhiping Deng

**Affiliations:** ^1^State Key Laboratory for Managing Biotic and Chemical Threats to the Quality and Safety of Agro-Products, Institute of Virology and Biotechnology, Zhejiang Academy of Agricultural Sciences, Hangzhou, China; ^2^Zhejiang Key Laboratory of Digital Dry Land Crops, Institute of Crops and Nuclear Technology Utilization, Zhejiang Academy of Agricultural Sciences, Hangzhou, China

**Keywords:** seeds, quantitative proteomics, oil content, rapeseed, *Brassica napus*, LEA protein, late embryogenesis abundant protein, TMT

## Abstract

Enhancing oil content is one of the major goals in *Brassica napus* breeding; however, genetic regulation of seed oil content in plants is complex and not fully elucidated. In this study, we report proteins that were differentially accumulated in immature seeds of 35 days after anthesis between two recombinant inbred lines with contrasting seed oil content, high oil content line (HOCL) and low oil content line (LOCL) using a multiplex isobaric tandem mass tags (TMT)-based quantitative proteomic approach. Over 4,600 proteins were quantified in seeds of the two lines, and 342 proteins showed differential accumulation between seeds of HOCL and LOCL. Gene Ontology enrichment analysis revealed that the differentially accumulated proteins were enriched in proteins involved in lipid biosynthesis and metabolism, photosynthesis, and nutrient reservoir activity. Western blot confirmed the increased abundance of a late embryogenesis abundant protein (BnLEA57) in HOCL seeds compared with LOCL seeds, and overexpression of either *BnLEA57* gene or its homology *BnLEA55* in transgenic *Arabidopsis thaliana* enhanced oil content in *Arabidopsis* seeds. Our work provides new insights into the molecular regulatory mechanism of seed oil content in *B. napus*.

## Introduction

Rapeseed (*Brassica napus* L.) is an important oil-producing crop in the world because of large demand for both edible and industrial purposes (Dupont et al., 1989). Furthermore, *B. napus* oil has been pursued as an attractive alternative for renewable biofuels (Sharma et al., 2015). Improving seed oil content in *B. napus* is still one of the most important breeding objectives (Hua et al., 2016).

*Brassica napus* seeds accumulate oils during seed development, and the storage of seed oil is mainly in the embryo in the form of triacylglycerol (TAG) (Bai et al., 2020). The biosynthetic pathway of the fatty acid and TAG in higher plants has been well elucidated, including in *Arabidopsis thaliana*, *Glycine max*, and *Brassica napus* (Maeo et al., 2009; Woodfield et al., 2019; Chen et al., 2020); however, the regulatory mechanism of seed oil accumulation is only partly understood. Modification of key genes involved in fatty acid biosynthesis, lipid biosynthesis, or glycolysis is known to affect seed oil accumulation (Hua et al., 2016; Herman, 2017). Several transcriptional factors that include *Leafy Cotyledon1* (*LEC1*) (Mu et al., 2008), *Leafy Cotyledon2* (*LEC2*) (Stone et al., 2008), *ABA-Insensitive3* (*ABI3*) (Giraudat et al., 1992), *FUSCA3* (*FUS3*) (Wang and Perry, 2013), *Transparent Testa2* (*TT2*) (Wang et al., 2014), and *Wrinkled1* (*WRI1*) (Liu et al., 2010) were shown to regulate lipid biosynthesis and seed oil accumulation. In addition, a few studies have demonstrated the maternal effects on seed oil content, such as *Apetala2* (*AP2*) (Jofuku et al., 2005) and *Transparent Testa8* (*TT8*) (Chen et al., 2014) in *Arabidopsis*.

Molecular genetic studies have revealed a large number of loci involved in controlling *B. napus* oil content, but the identification of candidate genes from QTLs controlling seed oil content is rare because of the presence of large intervals and small effect of each QTL on oil accumulation (Rahman et al., 2013; Hua et al., 2016). The omics strategy such as transcriptome studies has identified a large number of genes associated with seed oil accumulation (Weselake et al., 2009; Tan et al., 2011; Huang et al., 2017; Tang et al., 2021). By combing comprehensive genome- and transcriptome-wide association studies using 505 inbred lines, Tang et al. (2021) identified two seed-oil-content QTLs as two homologous putative methyltransferase *BnPMT6*s and validated their functions using genetic studies on CRISPR/Cas9 mutants of each gene.

Seed oil accumulation in *B. napus* is accomplished by a complex array of physiological and biological processes, and members of late embryogenesis abundant (LEA) proteins are associated in this process. There are three main stages during seed oil accumulation in *B. napus*. The first stage is endosperm development. At this stage, considerable soluble sugars accumulate in the developing seeds mainly for osmotic regulation for seed expansion, substrate preparation for oil and protein biosynthesis, and signaling molecules for oil and other secondary metabolism (Lorenz et al., 2014). The second stage is embryo development. Approximately 20 days after anthesis, the embryo initiates, and then, the endosperm is quickly and thoroughly absorbed by the embryo. Then, the seed is full of the embryo and a layer of seed coat surrounding the embryo (Schwender and Ohlrogge, 2002). Most of the seed storages such as oil and proteins are mainly synthesized at this stage (Schwender and Hay, 2012). The third stage is seed dehydration and maturation. During seed maturation, considerable water in the seed will be dehydrated because excessive water will affect seed vitality. In *B. napus* seed, the oil does not exist as the formation of free fatty acids but in the form of oil body. The synthesized fatty acid normally is transported into endoplasmic reticulum membrane for further folding into oil body (He and Wu, 2009). The process is always accompanied by dehydration although the exact functions of dehydration are not very clear (Quettier and Eastmond, 2009). In previous investigations, a large family of small and highly hydrophilic proteins named late embryogenesis abundant (LEA) proteins was identified in plant tissues such as in maturating seeds (Liang et al., 2016, 2019). LEA proteins are mostly associated with the drought and freezing tolerance in plants (Liang et al., 2019) and are suggested to stabilize membranes and macromolecules by acting as water-binding molecules (Wang et al., 2003; Chakrabortee et al., 2012). But the exact functions of LEA proteins are largely unknown, especially during seed development and maturation in *B. napus*.

With the rapid improvement in the sensitivity, reproducibility and high throughput of liquid chromatography with tandem mass spectrometry (LC-MS/MS) -based quantitative proteomics, quantitative proteomics has become an important tool in identifying candidate proteins in improving plant productivity and stress responses (Li et al., 2011; Hu et al., 2015; Zhao et al., 2017; Wu et al., 2021; Zhu et al., 2022). In this study, TMT-based quantitative proteomics was used to uncover differentially accumulated proteins (DAPs) between two recombinant breeding lines differing in oil content, and an LEA protein BnLEA57 was found to be about two-fold in abundance in the higher oil content line (HOCL) compared to the low oil content line (LOCL). The function of the *BnLEA57* gene and its close homolog *BnLEA55* gene in seed oil accumulation was further analyzed through the ectopic expression in *Arabidopsis thaliana*. The result shed new insights on elucidating the molecular function of the LEA proteins on the oil biosynthesis in *B. napus*.

## Results

### Identification of Differentially Accumulated Proteins in Developing Seeds Between High Oil Content Line and Low Oil Content Line

**FIGURE 1:**
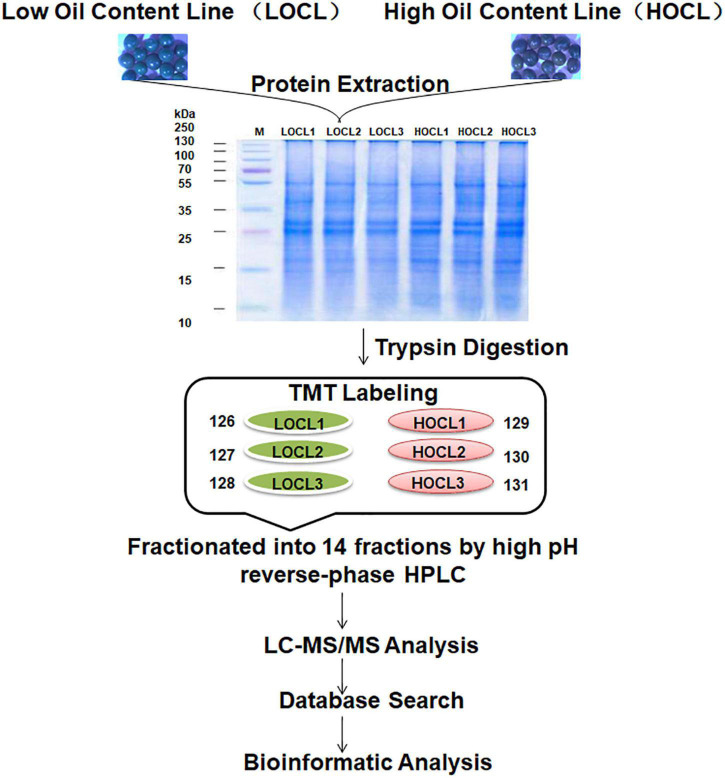
Workflow of TMT labeling-based proteome profiling in *B. napus* seeds. Total proteins were extracted from 35-DAA seeds of two recombinant inbred lines of the *B. napus* with high oil content (HOCL) and low oil content (LOCL) (each line with three biological repeats), digested with trypsin after protein quality was checked on SDS-PAGE. The peptides were individually labeled with 6-plex TMT reagent (LOCL: 126, 127, and 128 labels; HOCL: 129, 130, and 131 labels) before combined, fractionated, and analyzed by LC-MS/MS.

**FIGURE 2:**
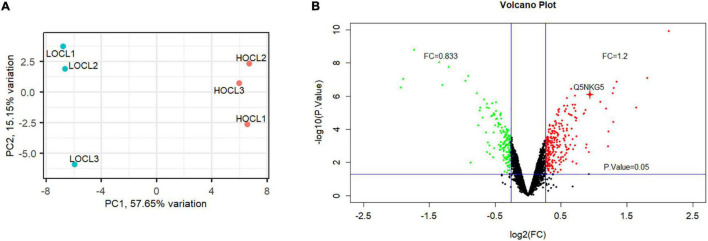
Principal component analysis and volcano plot of the proteome in seeds of HOCL and LOC. **(A)** Principal component analysis of the proteome in seeds of HOCL and LOCL. Percentages (%) indicate the percentage of overall variance captured by each principal component. PC1: principal component 1; PC2: principal component 2. **(B)** Volcano plot of DAPs between the seeds of HOCL and LOCL. Threshold cutoff (>1.2 or <0.83) determined for log2 fold change ratios are represented by two vertical blue lines. Adjusted *p*-value cutoff of 0.05 is represented by a horizontal blue line. Red dots represent up-accumulated proteins, and green dots indicate down-accumulated proteins, whereas back dots represent proteins that do not meet the threshold or adjusted *p*-value, in HOCL compared to LOCL. The red cross indicates BnLEA57 protein.

To identify DAPs in the developing seeds of HOCL and LOCL in *B. napus*, seeds at 35 days after anthesis (DAA), which were at the critical stage of seed oil accumulation (Hua et al., 2014), were sampled for proteomic analysis. Tandem mass tag (TMT)-based quantitation was used to compare the seed proteome between HOCL and LOCL, following the general workflow in Figure 1. A total of 5,106 *B. napus* proteins were identified with FDR at 1% protein level, and 4,638 proteins were quantified across the three biological replicates for both HOCL and LOCL (no imputation was performed) (Supplementary Table 1). The principal component analysis showed that the three replicates of HOCL and LOCL were clustered together, respectively, but both clusters were well separated in component I, indicating the reproducible differences present in the proteome between both lines (Figure 2A). The data of DAPs were further analyzed according to the fold change and adjusted *p*-value. The result of volcano plot (Figure 2B) showed that 342 proteins were significantly different between HOCL and LOCL with a fold change >1.2 or <0.83 and adjusted *p*-value less than 0.05. Among those proteins, there were 199 proteins up-accumulated and 143 proteins down-accumulated in the HOCL compared to the LOCL (Supplementary Table 1).

### Gene Ontology Enrichment Analysis of the Differentially Accumulated Proteins

**FIGURE 3:**
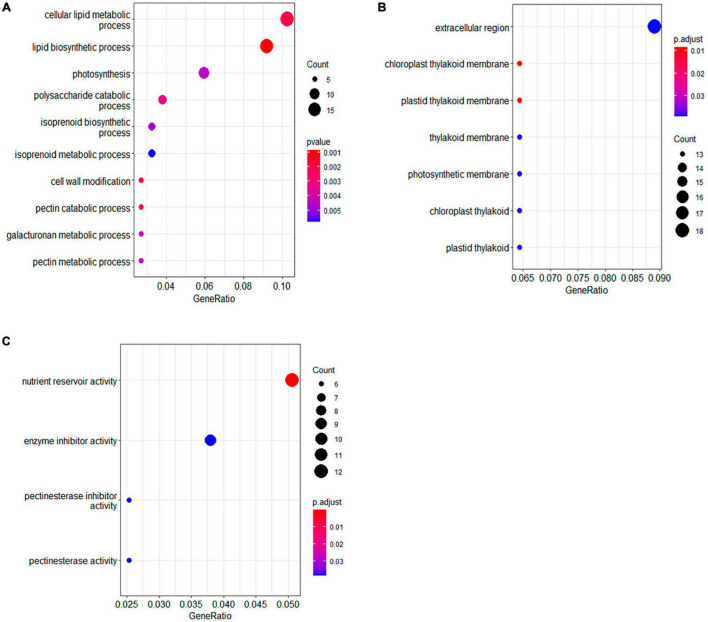
Gene Ontology enrichment analysis for the differentially accumulated proteins between HOCL and LOCL. Gene Ontology enrichment analysis was categorized according to biology process [BP, **(A)**], cellular component [CC, **(B)**], and molecular function [MF, **(C)**].

The functions of the DAPs were further analyzed by Gene Ontology (GO) enrichment. The DAPs were classified by GO terms according to three categories, namely, biological process, molecular function, and cellular component. In biological process, the largest functional enriched groups were lipid biosynthesis and metabolism (Figure 3A). Among lipid metabolic proteins, two biotin carboxyl carrier proteins of acetyl-CoA carboxylases (BCCPs) were significantly up-accumulated in the seeds of HOCL (Table 1). There were two acyl carrier proteins downregulated whereas one upregulated in the seed cells of HOCL. A protein encoding beta-ketoacyl-(acyl-carrier-protein) synthase (KAS) was also markedly upregulated (Table 1). Another important type of the DAPs was related to photosynthesis. The proteins were involved in the whole process of photosynthesis in the seed cells including antenna proteins (LHCA1 and LHCB5), photosystem II proteins (PsbE, PsbQ, and PsbR), photosystem I proteins (PsaL and PsaN), ATPase (AtpH), and ribulose bisphosphate carboxylase (RbcL). Importantly, all the proteins except two ribulose bisphosphate carboxylase small subunits were significantly higher in the seed cells of HOCL (Table 1). For the DAPs of essential components of the photosynthetic apparatus, all of the proteins involving in isoprenoid biosynthesis were upregulated except one zeta-carotene desaturase (Supplementary Table 2). Amazingly, all the DAPs associated with cell wall modification, the polysaccharide metabolism, pectin catabolic–metabolic process, and galacturonan metabolic process were the members of pectinesterase family, suggesting a role of cell wall modification on oil accumulation (Supplementary Table 2).

**TABLE 1:** Selected DAPs in immature seeds of 35DAA between HOCL and LOCL.

Accession	Description	Gene locus	GO description	Ratio	Adjusted *p-*value
A0A078E790	Acyl carrier protein	BnaAnng23710D	Lipid biosynthetic process	0.77	1.66E-02
A0A078FHD7	3-ketoacyl-CoA synthase	BnaA09g40640D	Lipid biosynthetic process	0.63	3.98E-04
A0A078FXF0	Transferase activity, transferring alkyl or aryl	BnaA06g35250D	Lipid biosynthetic process	1.22	1.44E-02
A0A078G4T7	Acyl carrier protein	BnaA09g03610D	Lipid biosynthetic process	1.24	1.29E-02
A0A078GDQ4	Hypothetical protein	BnaA05g30440D	Lipid biosynthetic process	1.20	1.22E-02
A0A078HJT1	Biotin carboxyl carrier protein of acetyl-CoA carboxylase	BnaC02g06560D	Lipid biosynthetic process	1.45	5.78E-03
A0A078HL12	Alkyl transferase	BnaC03g26600D	Lipid biosynthetic process	1.22	3.36E-02
A0A078HTG3	Phosphoethanolamine N-methyltransferase activity	BnaC06g02100D	Lipid biosynthetic process	0.80	7.71E-03
A0A078HTK0	Zeta-carotene desaturase	BnaC05g46990D	Lipid biosynthetic process	0.83	1.19E-02
A0A078HX24	Glycerol-3-phosphate acyltransferase, chloroplastic	BnaC05g28890D	Lipid biosynthetic process	0.83	3.18E-02
A0A078I1J8	Beta-ketoacyl-[acyl-carrier-protein] synthase I	BnaA02g24400D	Lipid biosynthetic process	1.65	9.10E-04
A0A078IU40	Xanthoxin dehydrogenase	BnaA06g01590D	Lipid biosynthetic process	1.57	1.33E-04
A0A078JFC7	Diphosphomevalonate decarboxylase	BnaCnng46900D	Lipid biosynthetic process	1.29	1.78E-02
A0A0K0K5D2	Zeta-carotene desaturase	BnaAnng10690D	Lipid biosynthetic process	2.55	7.34E-05
G4WXD9	Biotin carboxyl carrier protein of acetyl-CoA carboxylase	BnaC03g07000D	Lipid biosynthetic process	1.24	2.90E-02
P10352	Acyl carrier protein, chloroplastic, ACL1.A1	BnaA09g03610D	Lipid biosynthetic process	0.65	6.12E-04
Q8GV01	Lipoxygenase	BnaC06g26190D	Lipid biosynthetic process	0.80	5.67E-03
A0A078F5L4	Protein RALF-like 33	BnaC07g33210D	Extracellular region	0.79	1.76E-02
A0A078FGJ4	Mannan endo-1,4-beta-mannosidase	BnaA03g00090D	Extracellular region	0.80	1.06E-02
A0A078FNY0	FAD-binding PCMH-type	BnaA09g28890D	Extracellular region	1.32	5.78E-03
A0A078FSV0	FAD-binding PCMH-type	BnaA09g28900D	Extracellular region	1.36	1.20E-03
A0A078G1F3	Peroxidase OS = *Brassica napus*	BnaC01g31810D	Extracellular region	0.75	1.20E-03
A0A078GK30	Peroxidase OS = *Brassica napus*	BnaA04g12860D	Extracellular region	1.27	1.02E-02
A0A078GRW5	Carboxypeptidase	BnaA01g06330D	Extracellular region	0.80	4.57E-02
A0A078GYJ4	S-protein homolog	BnaA04g15920D	Extracellular region	1.29	1.08E-02
A0A078H673	(Rape) hypothetical protein	BnaC03g27700D	Extracellular region	0.62	1.60E-03
A0A078HGM0	Dirigent protein	BnaC01g37520D	Extracellular region	0.75	1.70E-03
A0A078HT58	(Rape) hypothetical protein	BnaC05g14950D	Extracellular region	1.37	1.83E-02
A0A078I5K0	Ribonuclease T(2)	BnaA06g33900D	Extracellular region	1.29	2.12E-02
A0A078IG99	Cysteine-type peptidase	BnaA06g16650D	Extracellular region	1.47	9.10E-04
A0A078IRK4	S-protein homolog	BnaA07g37790D	Extracellular region	1.30	2.47E-02
A0A078ITV4	Fn3_like domain-containing protein	BnaC06g27770D	Extracellular region	1.39	2.01E-02
A0A078J4F0	Pectin acetylesterase	BnaA04g27440D	Extracellular region	1.21	1.86E-02
A0A078JZ92	Gibberellin-regulated protein 8	BnaCnng75550D	Extracellular region	1.42	3.05E-02
Q2MD61	Rapeseed trypsin inhibitor-3, RTI-3	BnaC04g49020D	Extracellular region	0.26	1.24E-04
A0A078FMD5	2S SEED STORAGE PROTEIN 1-RELATED	BnaA08g14120D	Nutrient reservoir activity	1.50	3.22E-02
A0A078FMU9	11S globulin	BnaA08g13680D	Nutrient reservoir activity	1.31	4.17E-02
A0A078GA98	11S globulin	BnaA06g36310D	Nutrient reservoir activity	1.47	1.25E-02
A0A078GXC5	2S SEED STORAGE PROTEIN 1-RELATED	BnaC01g19300D	Nutrient reservoir activity	1.27	1.47E-02
A0A078IBF3	Napin/2S seed storage protein/Conglutin	BnaA03g48490D	Nutrient reservoir activity	1.64	6.37E-04
A0A078K0Y7	11S globulin	BnaAnng37930D	Nutrient reservoir activity	0.53	5.97E-05
P01090	Napin-2	BnaA01g16200D	Nutrient reservoir activity	1.21	3.48E-02
P17333	Napin, NAP1	BnaC01g19300D	Nutrient reservoir activity	1.33	1.03E-02
P33524	Cruciferin BnC2	BnaA02g22500D	Nutrient reservoir activity	0.66	1.45E-02
Q39344	*Brassica napus* napB napin	BnaA01g17200D	Nutrient reservoir activity	0.72	9.10E-04
Q7XB53	11S globulin (Fragment)	BnaC07g48660D	Nutrient reservoir activity	1.56	1.73E-02
Q9S9E6	Napin large chain L2B	BnaC01g19330D	Nutrient reservoir activity	1.23	3.77E-02
A0A075M3Q5	Ribulose bisphosphate carboxylase small subunit	BnaA02g12840D	Photosynthesis	0.82	3.96E-02
A0A078F3B2	Chlorophyll a-b binding protein, LHCA1	BnaC06g15470D	Photosynthesis	1.25	4.70E-02
A0A078HMF9	PSI subunit V, PsaL	BnaA04g07250D	Photosynthesis	1.25	3.36E-02
A0A078HR87	Photosystem I subunit PsaN	BnaC02g42890D	Photosynthesis	1.23	1.45E-02
A0A078IAV5	Photosystem II oxygen-evolving enhancer protein, PsbQ	BnaA09g19870D	Photosynthesis	1.27	1.03E-02
A0A078IFG6	F-type H+-transporting ATPase, AtpH	BnaA09g23090D	Photosynthesis	1.38	7.71E-03
A0A078J8I6	Chlorophyll a-b binding protein, LHCB5	BnaC02g46810D	Photosynthesis	1.60	1.14E-02
A0A078JXE7	Photosystem II 10 kDa polypeptide, PsbR	BnaAnng40550D	Photosynthesis	1.66	2.13E-03
A0A078JZM9	Ribulose bisphosphate carboxylase small subunit	BnaAnng36210D	Photosynthesis	0.83	2.77E-02
D1L8Q5	Cytochrome b559 subunit alpha, PsbE	BnaC04g29450D	Photosynthesis	1.62	7.83E-04
Q71SX0	Ribulose bisphosphate carboxylase large chain, RbcL	BnaA01g34300D	Photosynthesis	1.25	1.22E-02

*DAPs involved in lipid biosynthetic process, extracellular region, nutrient reservoir activity, and photosynthesis were included. Ratio indicates the average protein abundance ratio of HOCL/LOCL.*

For cellular component, GO-enrichment-identified terms include extracellular region and chlorophyll thylakoid membrane. DAPs associated with chlorophyll thylakoid membrane term were mostly involved in photosynthesis, and all the 13 DAPs were upregulated in HOCL (Supplementary Table 2). A total of 18 DAPs were extracellular region proteins, 11 of which were up-accumulated in HOCL. Functions of these extracellular region proteins in seed oil accumulation are mostly unexplored, which include trypsin inhibitor, pectin acetylesterase, and peroxidase.

For the molecular function terms, the largest enriched group in DAPs was the nutrient reservoir activity, which included 12 DAPs (Table 1), nine of which were up-accumulated in the HOCL. These proteins included seed storage proteins on the endoplasmic reticulum and are the members of the cruciferin (11S globulin) or napin (1.7–2S albumin) family. The second largest group was enzyme inhibitor activity which included five pectinesterase, three pectinesterase inhibitors, one Kunitz-type soybean trypsin inhibitor (STI), and one cysteine proteinase inhibitor, four of which showed up-accumulation in HOCL (Supplementary Table 2).

### Immunoblot Analysis of BnLEA57 in Developing Seeds of *B. napus*

**FIGURE 4:**
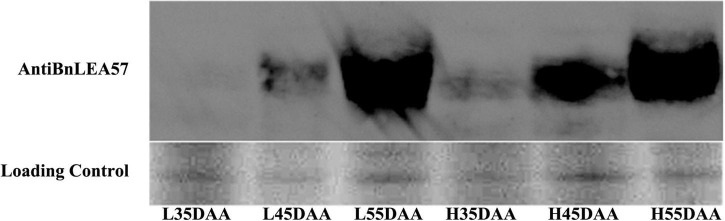
Protein abundance of BnLEA57 in different seed development stages and in different lines (HOCL and LOCL). Seed protein samples were probed with an anti-BnLEA57 antibody **(top lane)**. Coomassie blue-stained RuBisCO large subunit (RbcL) was used as a loading control **(bottom lane)**.

Among the DAPs, one late embryogenesis abundant protein, BnLEA57 (BnaC05g37670D), was chosen for further analysis because of its much higher abundance in the HOCL (the expression ratio between HOCL and LOCL was 1.91) and the implication of LEA proteins in dehydration stress response (Liang et al., 2016). Its accumulation in the developing seeds in the two lines was further examined by immunoblot analysis. The result showed that BnLEA57 protein abundance rose from 35 to 45 DAA and sharply increased from 45 to 55 DAA in the developing seeds of both lines. In addition, BnLEA57 protein accumulation in 35- and 45-, DAA seeds was consistently higher in HOCL than in LOCL (Figure 4), supporting the proteomic analysis results.

### Overexpression of the *BnLEA57* and *BnLEA55* Increased Seed Oil Content in *Arabidopsis*

**FIGURE 5:**
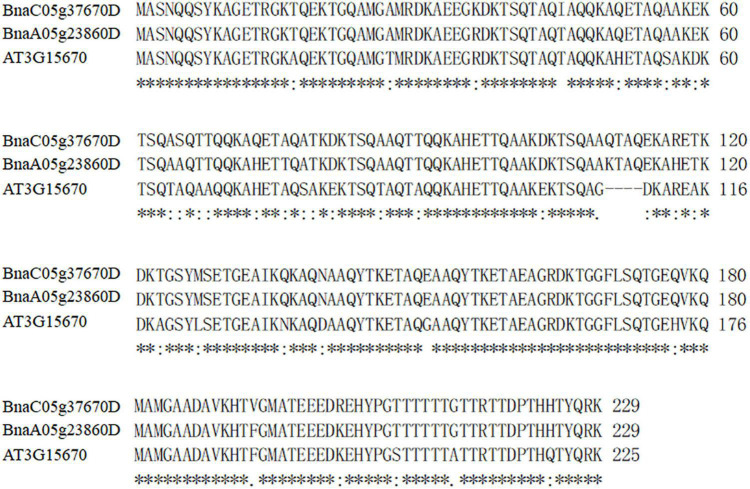
Alignment of the amino acid sequences of BnLEA57 (BnaC05g37670D), BnLEA55 (BnaA05g23860D) and their *Arabidopsis* ortholog AtLEA76 (AT3G15670). Residues identical in all the three LEAs are denoted with an asterisk (*), residues shared by two LEAs are indicated with a dot (.) or a colon (:).

**FIGURE 6:**
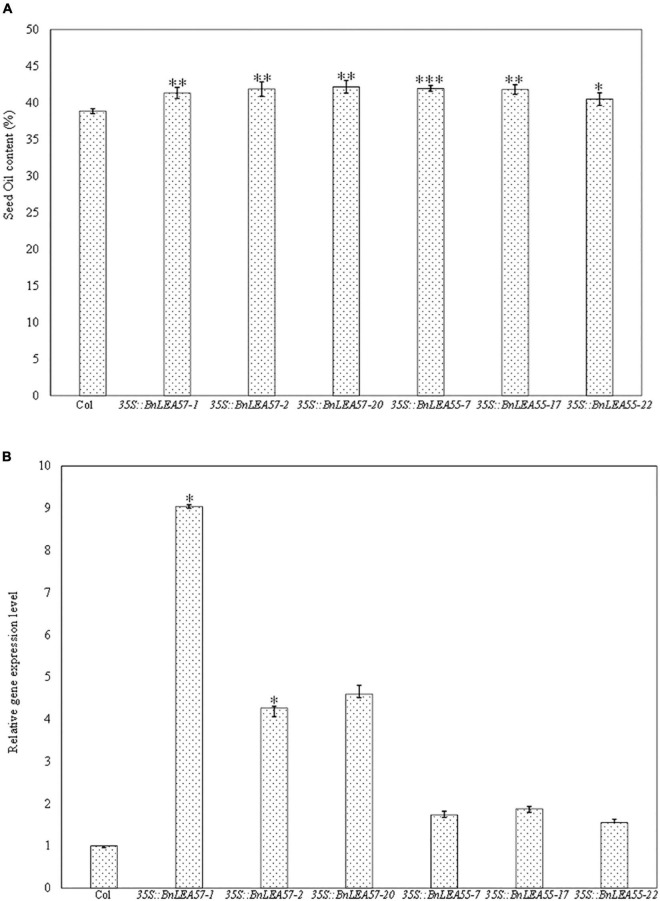
Overexpression of *BnLEA57* or *BnLEA55* gene in *Arabidopsis* increased seed oil content. **(A)** Seed oil content in the Col-0 (WT) and different transgenic lines overexpressing *BnLEA57* or *BnLEA55 gene.* **(B)** Relative gene expression level of *BnLEA57* and *BnLEA55* in different *Arabidopsis* transgenic lines. Error bars indicate standard deviation (*n* = 3). Significant difference between the transgenic plants and wild type (Col-0) is indicated: * for *p* < 0.05, ^**^ for *p* < 0.01, and ^***^ for *p* < 0.001 for Student’s t-test significance.

To further characterize the *BnLEA57* function on seed oil deposition, we overexpressed *BnLEA57* (*BnaC05g37670D*) and its closest *B. napus* homolog *BnLEA55* (*BnaA05g23860D*) in *Arabidopsis* plants, respectively. Less sequence similarity was observed among the 108 known LEA proteins in *B. napus* (Liang et al., 2016), but BnLEA57 and BnLEA55 that shared 96.1% of sequence identity (Figure 5) were clustered together in a subgroup in the LEA_4 family. BnLEA57 and BnLEA55 shared amino acid sequence identity of 84.3 and 86% with their ortholog LEA76 (AT3G15670) in *Arabidopsis*, respectively (Figure 5). Overexpression of either *BnLEA57* or *BnLEA57* in *Arabidopsis* increased seed oil content in *Arabidopsis*, which is consistent with the increased *LEA57*/*LEA57* transcript levels compared with wild type (Figures 6A,B). The mean oil content was 38.9% in wild type seeds, 41.8% in *35S::BnLEA57* seeds, and 41.4% in *35S::BnLEA55* seeds. The results revealed the positive regulation of seed oil content by both *BnLEA57* and *BnLEA55* genes.

## Discussion

Fatty acid biosynthesis and oil body formation in *B. napus* seeds are the intricate and complicated networks of physiological and biochemical processes, and the underlying molecular mechanism remains to be further unveiled and characterized. In this study, TMT-based quantitative proteomics was applied to identify the proteins mediating seed oil accumulation *via* a pair of *B. napus* lines with contrasting seed oil content. GO enrichment analysis of DAPs suggests that proteins involved in lipid metabolism, photosynthesis, and nutrient reservoir activity are associated with contrasting seed oil accumulation between HOCL and LOCL (Figure 3). In addition, a role of LEA protein BnLEA57 and its homolog BnLEA55 in seed accumulation was further supported by their ectopic expression in *Arabidopsis*.

Our proteomic study suggests that higher photosynthesis capacity in the seeds contributes to higher lipid biosynthesis and oil accumulation in HOCL. Previous physiological studies comparing HOCL with LOCL, the same inbred lines used in this study, indicate higher chlorophyll content in the siliques of HOCL, supporting that higher photosynthetic capacity in siliques contribute to increased oil content in HOCL seeds. Results from this proteomic study support that photosynthesis from seeds could also contribute to oil accumulation in seeds. Among the DAPs, upregulation of protein abundance of two photosynthesis marker genes (chlorophyll A/B-binding proteins) (Martin et al., 2002) and two fatty acid biosynthesis marker genes (BCCP) (Ruuska et al., 2002) were observed (Table 1). In addition, among DAPs, GO-enriched terms in biological process include photosynthesis (9 of 11 proteins were up-accumulated in HOCL), and the enriched terms in the cellular process include thylakoid membranes (of which 13 proteins were all up-accumulated in HOCL, also involved in photosynthesis), supporting that higher photosynthesis activity contributes to higher seed oil content in HOCL. During fatty acid biosynthesis, considerable carbohydrates as substrate are required. Although it was estimated that about 70% of the photoassimilates were from silique wall (King et al., 1998), the remains were from transportation from other tissues such as leaf and stem and from developing seeds itself. A few lines of evidence showed that photosynthetic organelles were observed and the photosynthetic capability was found in developing seed cells (Eastmond et al., 1996; King et al., 1998; Ruuska et al., 2004; Houston et al., 2009). In addition, photosynthesis in seeds could enhance seed oil biosynthesis by increasing the carbon utilization efficiency (Schwender et al., 2004; Hay and Schwender, 2011) and by providing adenosine triphosphate (ATP), reductants NADPH, and NADH, which are the co-factors required for biosynthesis of fatty acids in chloroplasts (Goffman et al., 2005).

Among those DAPs, we found a late embryogenesis abundant protein BnLEA57 that was up-accumulated in the HOCL and showed that BnLEA57 and its homolog BnLEA55 positively regulate seed oil accumulation. It is known that LEAs are involved in the abiotic stress such as water deficiency and salt resistance (Dalal et al., 2009; Cuevas-Velazquez et al., 2014), but the exact molecular mechanism is poorly understood. In *B. napus*, Liang et al. (2019) found that overexpression of members of LEA_3 family (*BnLEA3.1*, *BnLEA3.2*, *BnLEA3.3*, and *BnLEA3.4*) increased oil content in *Arabidopsis* and *B. napus* seeds and suggested that LEA proteins increase seed oil accumulation by enhancing photosynthetic efficiency and improving membrane stability. The enhancement of photosynthetic capacity may result in the increment of substrate for fatty acid biosynthesis, namely, carbohydrate. The result was in accordance with our proteomic analysis. The LEA_4 family proteins BnLEA57 and BnLEA55 in our experiment were also shown to improve seed oil content when overexpressed in *Arabidopsis*. There were 108 LEA genes identified in *B. napus* genome, and many of them showed increased expression at late-stage seeds (Liang et al., 2016). Further investigations are required to reveal the accurate molecular mechanism of BnLEA57 and BnLEA55 on seed oil accumulation and to characterize whether different members of LEA proteins work individually or together on seed maturity and oil accumulation.

## Materials and Methods

### Plant Materials and Sampling

A total of two recombinant inbred lines of *B. napus* with high oil content (50.4%, HOCL) and low oil content (41.4%, LOCL) lines, two recombinant inbred lines at the F8 generations derived from parental lines between Huyou 15 as male parents and Zheshuang 6 as female parents (Hua et al., 2012, 2014), were grown in the field on the campus of Zhejiang Academy of Agricultural Sciences as described (Hua et al., 2014). The seeds for western blot assay were collected at 35, 45, and 55 days after anthesis on the same position of main inflorescence, the samples were immediately frozen in liquid nitrogen and kept at –80°C until use, and the samples collected at 35 DAA were also used for proteomic analysis.

*Arabidopsis thaliana* ecotype Columbia-0 was used for transformation. *Arabidopsis* seeds were surface-sterilized with 20 mg g^–1^ NaClO solution for 10 min and washed five times by sterilized distilled water, sown on agar medium (half-strength Murashige and Skoog basal salt mixture, 15 g kg^–1^ sucrose, pH 5.7), and vernalized at 4°C for 3 days in the dark. After germination, seedlings were transferred into soil and then grown in a growth room at 20–22°C, 16-h/8-h (day/night) photoperiod, and 60–70% humidity. The seeds were harvested as matured for oil content analysis. A total of three biological replicates for each sample were taken for analysis.

### Protein Extraction and Digestion for Liquid Chromatography With Tandem Mass Spectrometry Analysis

Total proteins were extracted from the seeds of the *B. napus*, as previously described (Deng et al., 2007). Washed and pelleted protein sample was resuspended in 8 M urea solution, and the protein concentration was determined by the Bradford method.

For trypsin digestion, the samples were reduced with 10 mM DTT for 1 h at 56°C and alkylated with 55 mM iodoacetamide for 45 min at room temperature in darkness. Proteins were precipitated with 5 volumes of cold acetone overnight and then dissolved in 50 mM TEAB. Proteins were digested with trypsin overnight at 37°C in a 1:50 trypsin-to-protein mass ratio.

### Tandem Mass Tag Labeling and High Performance Liquid Chromatography Fractionation

After trypsin digestion, peptides were desalted by Strata X C18 SPE column (Phenomenex) and vacuum-dried. Peptides were reconstituted in 50 mM TEAB and then labeled with respective 6-plex TMT reagents (LOCL: 126, 127, and 128 tags; HOCL: 129, 130, and 131 tags). The samples were pooled and fractionated by high pH reversed-phase high performance liquid chromatography (HPLC) using Agilent 300Extend C18 column (5-μm particles, 4.6 mm ID, 250 mm length). Peptides were separated with a gradient of 2–60% acetonitrile in 10 mM ammonium bicarbonate (pH 8) over 80 min. The peptides were then combined into 14 fractions and dried by vacuum centrifuging.

### Liquid Chromatography With Tandem Mass Spectrometry Analysis

Multiplexed TMT-labeled samples were dissolved in 0.1% formic acid (solvent A), directly loaded onto a reversed-phase column (360 μm OD × 75 μm ID, 25 cm length) packed in-house with 3-μm C18 beads (ReproSil-Pur C18-AQ, Dr. Maisch) and eluted with a linear gradient of 6–25% solvent B (0.1% formic acid in 98% acetonitrile) for 28 min and 25–35% solvent B for 6 min at a constant flow rate of 300 nl/min on an EASY-nLC 1000 UPLC system. The resulting peptides were analyzed by Q Exactive Plus hybrid Quadrupole-Orbitrap mass spectrometer (Thermo Fisher Scientific, Waltham, MA, United States). A data-dependent procedure that alternated between one MS scan at a resolution of 70,000 and AGC target 3E6 followed by 20 MS/MS scans was applied for the top 20 precursor ions above a threshold ion count of 5E4 in the MS survey scan with 30.0-s dynamic exclusion. For MS scans, the m/z scan range was 350–1,600 Da. MS/MS scans were performed at the resolution at 17,500 and NCE at 30 with 3% stepped energy. The fixed first mass was set at 100 m/z for TMT quantification.

### Data Analysis

The MS/MS raw data were analyzed using Proteome Discoverer (Version 2.4.0.305, Thermo Fisher Scientific, Waltham, MA, United States). Peptide and protein identifications were performed using both SEQUEST and MSFragger search engines against *Brassica napus* proteome from UniprotKB database (total 62,904 entries, as of 8 August 2021). Both searches were performed using the following settings: enzyme as trypsin/P, carbamidomethyl (C), TMT6plex (N-term), and TMT6plex (K) were selected as fixed modifications and oxidation (M) as variable modifications, 2 missed cleavage allowed and precursor error tolerance at 10 ppm, fragment deviation at 0.02 Da. Principal component analysis (PCA) of the samples was performed with R package PCAtools^1^. R package limma was used in screening the differentially accumulated proteins (DAPs) between HOCL and LOCL (Phipson et al., 2016). Adjusted *p*-value < 0.05 and fold change >1.20 or <0.83 were chosen as the cutoff threshold. The volcano plot was plotted using EnhancedVolcano package from R^2^. Gene Ontology (GO) enrichment for proteins that met differential expression criteria was performed using clusterProfiler in R environment (Wu et al., 2021).

### Protein Immunoblot Analysis

Immunoblot analysis was performed as described (Deng et al., 2014). The proteins were extracted and dissolved in 2-D DIGE buffer (6 M urea, 2 M thiourea, and 4% CHAPS) and quantified using the Bio-Rad protein assay. Then, proteins were electrophoresed on 12% SDS-PAGE gels in a Tris-glycine buffer system at a constant 120 V/gel. Proteins were transferred to a nitrocellulose membrane at room temperature in a Bio-Rad semidry transfer System (Bio-Rad Laboratories, Hercules, CA, United States) according to the manufacturer’s instructions. The transferred membrane was immunoblotted with anti-BnLEA57 antibody (Abmart, Shanghai, China) and then incubated with goat-anti-rabbit IgG-horseradish peroxidase (HRP) secondary antibody before development with ECL SuperSignal West Dura Extended Duration Substrate (Thermo Fisher Scientific, Waltham, MA, United States), and the intensities were captured by the ImageQuant LAS 4000 mini system (GE Healthcare Life Sciences, Piscataway, NJ, United States). Anti-BnLEA57 antibody was produced using the synthetic peptide C-TTQQKAQETAQATK (Abmart, Shanghai, China).

### Vector Construction and Plant Transformation

cDNA sequences of *BnLEA57* (*BnaC05g37670D*) and *BnLEA55* (*BnaA05g23860D*) were downloaded from the *Brassica napus* genome database^3^. For both cloning constructs (*35S::LEA57* and *35S::LEA55*), target sequences were first cloned into the pENTR/D-TOPO vector (Invitrogen, Carlsbad, CA, United States) before being recombined into an destination vector pB7WGF2 using the gateway LR reaction (Invitrogen, Carlsbad, CA, United States). The following primers were used: LEA57-F, 5′-CACCATGGCGTCTAACCAACAGAGC-3′; LEA57-R, 5′-CC TCTGATAAGTATGATGAGTAGGA-3′; LEA55-F, 5′-CACCA TGGCGTCCAACCAACAA-3′; and LEA55-R, 5′-CTTCCTCTG ATAAGTATGATGAGTCG-3′. Genetic transformation was performed as described (Zhou et al., 2011). The plasmids were transformed into *Agrobacterium tumefaciens* GV3101 strain and then transformed into Columbia-0 genotype using the floral-dip method (Clough and Bent, 1998). Transgenic seedlings were first selected on agar growth media with appropriate antibiotics and then confirmed by PCR using the corresponding primers.

### RNA Extraction and Real-Time PCR Quantification

Total RNAs were extracted from the 10-day-old *Arabidopsis* seedlings with TRIzol Reagent (Invitrogen, Carlsbad, CA, United States), and cDNA was synthesized from 2 μg of total RNA using Moloney murine leukemia virus reverse transcriptase (Promega) and random primers in a 15 μl reaction according to the manufacturer’s instructions. For real-time PCR, the cDNAs were diluted to 4 times, and 1 μl of the cDNAs was added to 5 μl of PowerUP SYBR Green PCR Master Mix (Thermo Fisher Scientific, Waltham, MA, United States) and 0.4 μl of each primer (100 nM final concentration) in 10 μl reactions. PCR amplification and detection were performed using a Bio-Rad CFX96 Tough Real-time PCR Detection System using the following cycling conditions: 50°C for 2 min, 95°C for 2 min, followed by 40 cycles of 95°C for 15 s, and 60°C for 1 min. Melting-curve analysis was used to confirm the absence of non-specific amplification products. *UBQ5* transcripts were used as an endogenous control to normalize expression of other genes. Relative expression levels were calculated by subtracting the threshold cycle (Ct) values for *UBQ5* from those of the target gene and then calculating 2^–ΔCt^ as described previously (Zhou et al., 2011). RT-PCR experiments were performed on at least three independent samples. The following primers were used: UBQ5-F, 5′-TCGACGCTTCATCTCGTCCT-3′; UBQ5-R, 5′-CGCTGAACCTTTCCAGATCC-3′; LEA57-QPCR-F, 5′-ACAAAGGAGACGGCTCAAGA-3′; LEA57-QPCR-R, 5′-TTCCAACAGTGTGCTTCACC-3′; LEA55-QPCR-F, 5′-ACAAAGGAGACGGCTCAAGA-3′; LEA55-QPCR-R, 5′-GGTCGTAGTTGTGCCTGGAT-3′.

### Oil Content Measurement and Statistical Analysis

Oil content in *B. napus* seeds was determined as described (Hua et al., 2014). In this experiment, statistical analysis was carried out using two-tailed Student’s *t*-test with two-samples unequal variance (“*,” “^**^,” and “^***^” represents as *p* < 0.05, *p* < 0.01, and *p* < 0.001) for significance.

## Data Availability Statement

The datasets presented in this study can be found in online repositories. The names of the repository/repositories and accession number(s) can be found below: ProteomeXchange, accession number PXD033185.

## Author Contributions

ZD, SH, and ZZ designed the experiment and wrote the manuscript. ZZ, ZD, and JT performed the proteomic analysis including data analysis. ZZ performed the western blotting and created the transgenic *Arabidopsis* lines. SH, BL, and PH prepared the *B. napus* samples and analyzed the seed oil content. All authors contributed to the article and approved the submitted version.

## Conflict of Interest

The authors declare that the research was conducted in the absence of any commercial or financial relationships that could be construed as a potential conflict of interest.

## Publisher’s Note

All claims expressed in this article are solely those of the authors and do not necessarily represent those of their affiliated organizations, or those of the publisher, the editors and the reviewers. Any product that may be evaluated in this article, or claim that may be made by its manufacturer, is not guaranteed or endorsed by the publisher.

## Footnotes

1
https://github.com/kevinblighe/PCAtools

2
https://github.com/kevinblighe/EnhancedVolcano

3
https://plants.ensembl.org/Brassica_napus/Info/Index

## Supplementary Material

Supplementary Table 1 Lists of identified, quantified, and differentially accumulated *Brassica napus* proteins between seeds of HOCL and LOCL.

Supplementary Table 2 Functional protein groups identified by GO enrichment analysis of DAPs between HOCL and LOCL seed proteome.
