# Supplementary figures and images for: Tandem Mass Tag-Based Quantitative Proteomics Reveals Implication of a Late Embryogenesis Abundant Protein (BnLEA57) in Seed Oil Accumulation in Brassica napus L
Source: Front Plant Sci. 2022 Jun 2;13:907244. doi: 10.3389/fpls.2022.907244 (PMC9201403; doi:10.3389/fpls.2022.907244)

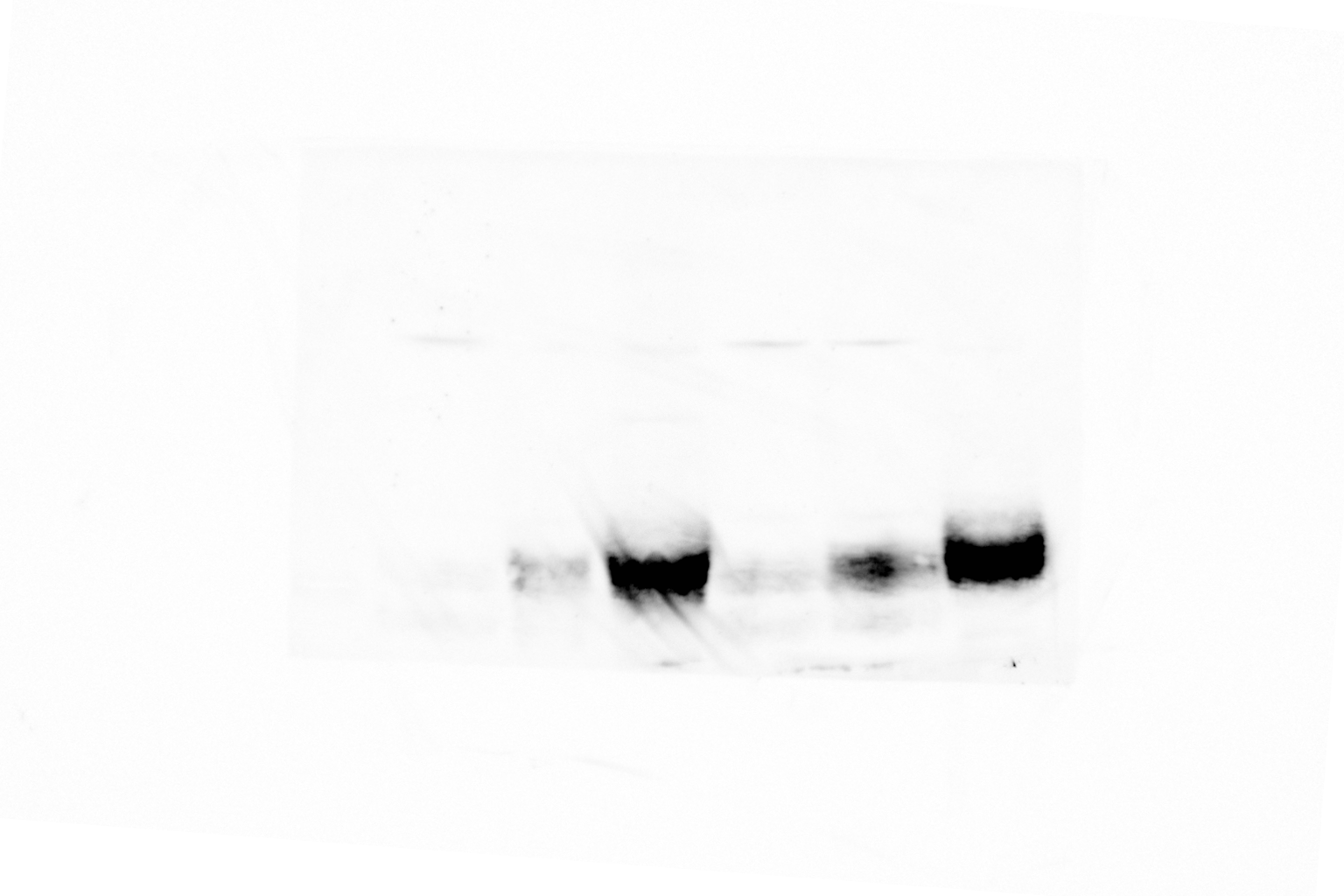

Supplement: Supplementary file 1 [file Data_Sheet_1.ZIP › LEA3_westernblot.tif]

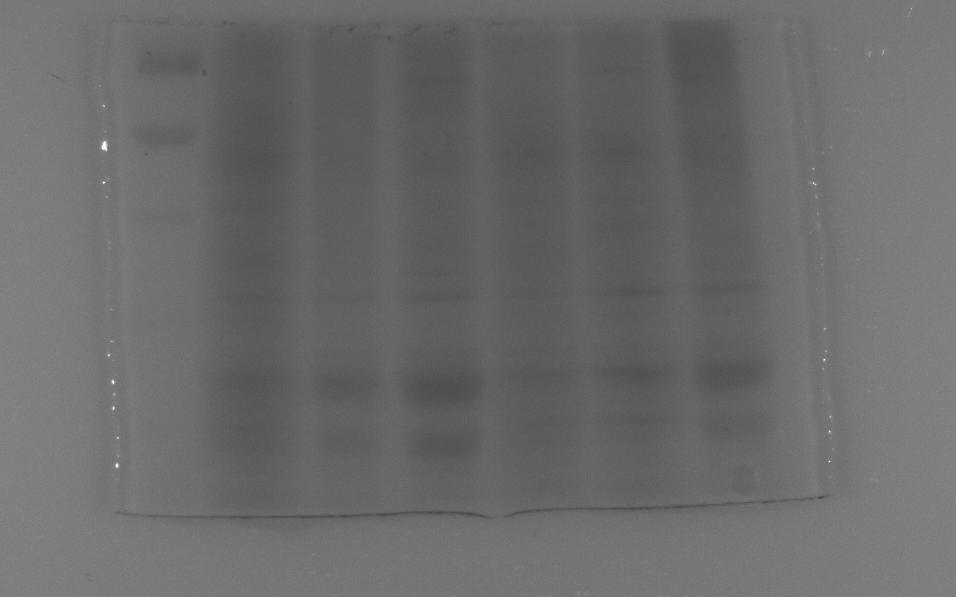

Supplement: Supplementary file 1 [file Data_Sheet_1.ZIP › Westernblot_loading_20220406.tif]
